# The node-weighted Steiner tree approach to identify elements of cancer-related signaling pathways

**DOI:** 10.1186/s12859-017-1958-4

**Published:** 2017-12-28

**Authors:** Yahui Sun, Chenkai Ma, Saman Halgamuge

**Affiliations:** 1 Department of Mechanical Engineering, The University of Melbourne, Melbourne, 3010 Australia; 2 Department of Surgery, The University of Melbourne, Melbourne, 3010 Australia; 3 Research School of Engineering, College of Engineering & Computer Science, The Australian National University, Canberra, 2601 ACT Australia

**Keywords:** Systems biology, Bioinformatics, Data mining, Big data

## Abstract

**Background:**

Cancer constitutes a momentous health burden in our society. Critical information on cancer may be hidden in its signaling pathways. However, even though a large amount of money has been spent on cancer research, some critical information on cancer-related signaling pathways still remains elusive. Hence, new works towards a complete understanding of cancer-related signaling pathways will greatly benefit the prevention, diagnosis, and treatment of cancer.

**Results:**

We propose the node-weighted Steiner tree approach to identify important elements of cancer-related signaling pathways at the level of proteins. This new approach has advantages over previous approaches since it is fast in processing large protein-protein interaction networks. We apply this new approach to identify important elements of two well-known cancer-related signaling pathways: PI3K/Akt and MAPK. First, we generate a node-weighted protein-protein interaction network using protein and signaling pathway data. Second, we modify and use two preprocessing techniques and a state-of-the-art Steiner tree algorithm to identify a subnetwork in the generated network. Third, we propose two new metrics to select important elements from this subnetwork. On a commonly used personal computer, this new approach takes less than 2 s to identify the important elements of PI3K/Akt and MAPK signaling pathways in a large node-weighted protein-protein interaction network with 16,843 vertices and 1,736,922 edges. We further analyze and demonstrate the significance of these identified elements to cancer signal transduction by exploring previously reported experimental evidences.

**Conclusions:**

Our node-weighted Steiner tree approach is shown to be both fast and effective to identify important elements of cancer-related signaling pathways. Furthermore, it may provide new perspectives into the identification of signaling pathways for other human diseases.

## Background

Cancer is a collection of diseases characterized by uncontrolled growth and spread of abnormal cells. It constitutes a major health burden in our society. For example, in 2012, approximately 14.1 million new cancer cases were diagnosed globally, and 8.2 million deaths or 14.6% of human deaths were caused [1]. Even though a large amount of money has been spent on cancer research [2], cancer-related signaling pathways have not been completely understood to date [3]. Hence, new works towards a complete understanding of cancer-related signaling pathways are highly recommended.

Some signaling pathways are already known to be cancer-related [4, 5]. Nevertheless, these existing signaling pathways may not be complete. Furthermore, most of them are recorded and analyzed at the level of genes and genomes, while that at the level of proteins have so far been rarely explored, although critical information may be hidden in them. In this work, we aim to identify important elements of cancer-related signaling pathways at the level of proteins.

There are mainly three types of approaches to identify signaling pathways, which are the experimental approach [6], the systematic approach [7], and the data-driven approach [8–11]. The experimental approach identifies signaling pathways by discovering biomedical evidences through experiments; the systematic approach identifies signaling pathways by integrating biomedical experiments with data analysis techniques; the data-driven approach identifies signaling pathways by purely processing previous biomedical data. All the three approaches have been successfully applied to identify signaling pathways for various human diseases. However, due to the slowness of experiments in the experimental and systematic approach, the data-driven approach may be the only one that is fast in large networks.

Protein-protein interaction networks are often very large. Therefore, it may be preferable to use the data-driven approach to identify cancer-related signaling pathways at the level of proteins. The Steiner tree approach is an efficient data-driven approach that has been applied to process biomedical data [12–14]. It can identify smaller subnetworks from large networks while keeping all the potentially important information, and investigators can then perform a more detailed, experimental-evidence-based analysis on these subnetworks. Thus, in this work, we use the Steiner tree approach to identify important elements of cancer-related signaling pathways.

There are different types of Steiner tree approaches. Researchers have already applied the classical Steiner tree approach [15] and the prize-collecting Steiner tree approach [16] to biomedical networks. However, as to protein-protein interaction networks, the classical Steiner tree approach fails to consider the properties of different proteins, while the prize-collecting Steiner tree approach may identify irrelevant proteins. Therefore, neither of them is suitable for processing protein-protein interaction networks. In this paper, we apply the node-weighted Steiner tree approach to protein-protein interaction networks for the first time. It advantages the classical Steiner tree approach and the prize-collecting Steiner tree approach since it considers the properties of different proteins by attaching them with node weights and it can avoid irrelevant proteins by attaching them with negative node weights.

The definition of node-weighted Steiner tree problem is given as follows: Let *G*=(*V*,*E*,*w*,*c*) be a connected, undirected network, where *V* is the set of vertices, *E* is the set of edges, *w* is a function which maps each vertex in *V* to a real number called the node weight, and *c* is a function which maps each edge in *E* to a positive number called the edge cost. Let *T*⊆*V* be a subset of *V* called compulsory terminals. The purpose of this problem is to find a connected subnetwork *G*′=(*V*′,*E*′),*T*⊆*V*′⊆*V*,*E*′⊆*E* which minimizes the objective function $c(G{\prime })=\sum _{e \in E{\prime }}{c(e)}-\sum _{v \in V{\prime }}{w(v)}$. In our application to protein-protein interaction networks, vertices represent proteins, edges represent protein-protein interactions, compulsory terminals represent important proteins to cancer signal transduction, edge costs represent in-confidence scores of the existence of protein-protein interactions, and node weights represent confidence scores of the existence of proteins in cancer-related signaling pathways. Under these representations, we can identify subnetworks containing important elements of cancer-related signaling pathways by solving the node-weighted Steiner tree problem.

**Fig. 1:**
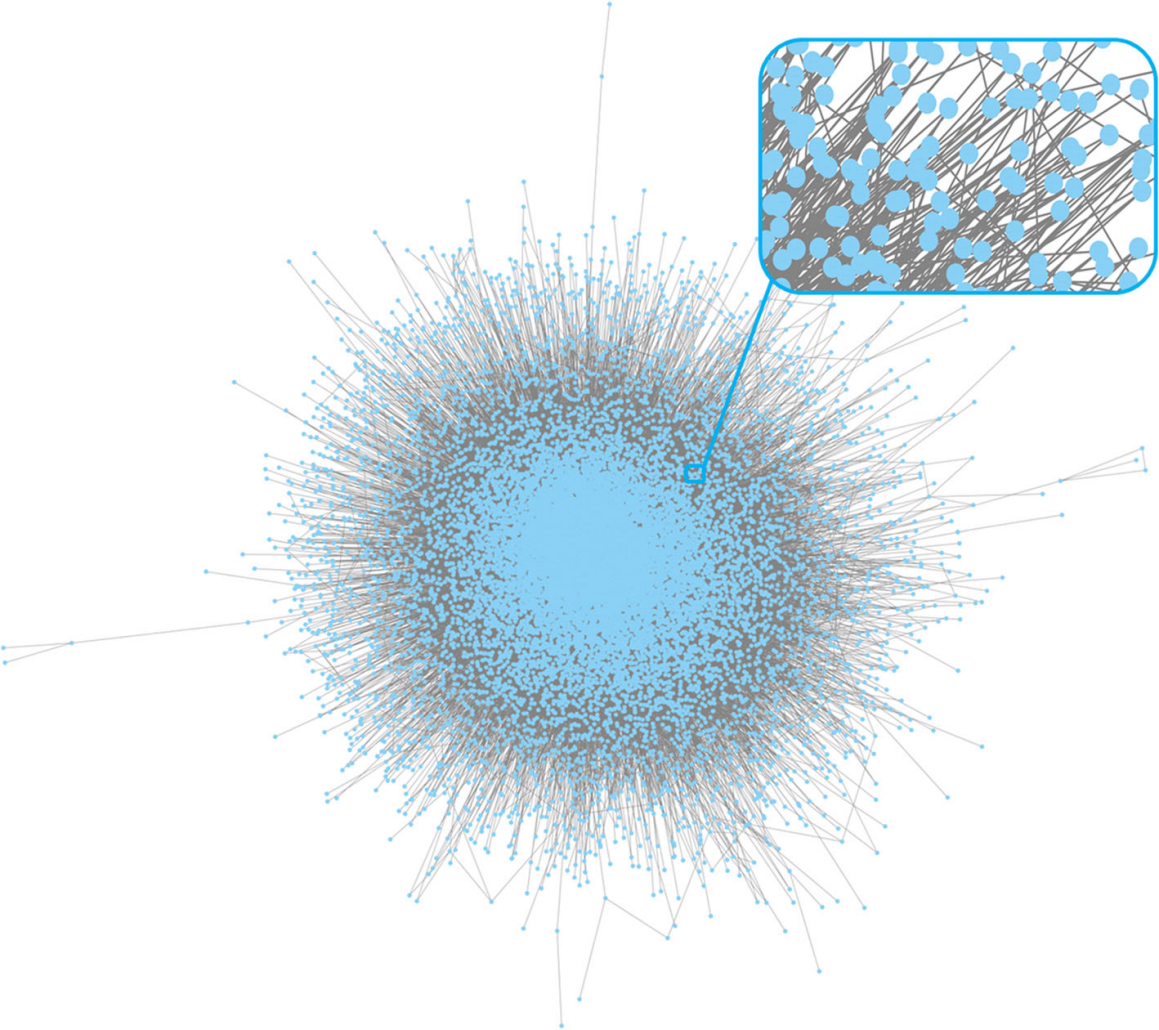
Topology of the generated node-weighted protein-protein interaction network for Homo sapiens. Each blue dot represents a protein, and each gray line represents a protein-protein interaction. There are 16,843 vertices and 1,736,922 edges in total

Nevertheless, it is still challenging to solve the node-weighted Steiner tree problem at present. Most existing techniques can only solve special cases of this problem, such as the classical Steiner tree problem in graphs [17] and the prize-collecting Steiner tree problem [18], while the ones that can solve the general node-weighted Steiner tree problem may be too slow in large protein-protein interaction networks [19]. Two types of Steiner tree techniques can deal with large networks efficiently. One is preprocessing technique, and the other one is heuristic algorithm. Therefore, in this work, we first modify two preprocessing techniques to reduce sizes of node-weighted Steiner tree instances. Then, we modify a state-of-the-art algorithm for the prize-collecting Steiner tree problem [20] to solve the general node-weighted Steiner tree problem. Our modified algorithm is fast in large networks. For instance, on a commonly used personal computer with a 4.2 GHz i7-7700K CPU, our modified algorithm only takes 0.05 second to identify a subnetwork in our generated large protein-protein interaction network for Homo sapiens (see Fig. 1), which has 16,843 vertices and 1,736,922 edges. Therefore, our modified algorithm can be applied to areas where fast processing of large protein-protein interaction networks is required.

The subnetwork identified by our node-weighted Steiner tree techniques contains important elements of cancer-related signaling pathways. It is necessary to select these important elements from the subnetwork for a further, more detailed analysis. There are many metrics to evaluate the importance of network elements [21, 22], among which betweenness centrality [23] is probably the most popular one. However, the original betweenness centrality fails to consider different functions of proteins in cancer-related signaling pathways. Thus, we propose new metrics that overcome this weakness to evaluate the importance of proteins and protein-protein interactions in the identified subnetwork. The important ones are then selected as the identified important elements of cancer-related signaling pathways.

In summary, our main contributions are as follows: we propose a method to generate protein-protein interaction networks with both positive and negative node weights; we modify two preprocessing techniques and a state-of-the-art heuristic algorithm to identify subnetworks in them; we propose two new metrics to select important elements of cancer-related signaling pathways from the identified subnetworks; we apply our node-weighted Steiner tree approach to identify important elements of two well-known cancer-related signaling pathways: PI3K/Akt and MAPK; we conduct an experimental-evidence-based analysis on the identified important elements, and a deeper understanding towards these two signaling pathways is gained in this process.

## Methods

### Generation of the node-weighted protein-protein interaction network

Protein-protein interaction networks are often very large, and critical information on cancer is hidden in them. In this section, we propose a method to generate node-weighted protein-protein interaction networks for the identification of important elements of cancer-related signaling pathways. We define a node-weighted protein-protein interaction network as a connected network with the following five types of elements:

**∙ vertex**: each vertex represents a protein.

**∙ edge**: each edge represents a protein-protein interaction.

**∙ compulsory terminal**: each compulsory terminal represents a protein that must be contained in the identified subnetwork. Since the purpose is to identify important elements of cancer-related signaling pathways, proteins that are well known to be important to cancer signal transduction are selected to be compulsory terminals.

**∙ edge cost**: edge cost is a positive value attached to each edge. Since the node-weighted Steiner tree technique tends to minimize the total edge cost in the identified subnetwork, we use edge costs to represent in-confidence scores of the existence of protein-protein interactions. As a result, the identified subnetwork tends to contain the most credible protein-protein interactions for cancer signal transduction. The quantified edge cost is calculated using the equation below,

1 $$ c(i,j)= \frac{\alpha}{con^ \beta}  $$

where *i* and *j* are indexes of two different proteins, *c*(*i*,*j*) is the cost of edge (*i*,*j*), *α*,*β* are positive constant values, and *con* is a score reflects the confidence of the existence of this protein-protein interaction.

**∙ node weight**: node weight is a real value attached to each vertex. The identified subnetwork tends to contain proteins with big positive node weights while avoid proteins with big negative node weights. Hence, we use node weights to represent confidence scores of the existence of proteins in cancer-related signaling pathways. The quantified node weight is calculated using the equation below,

2 $$\begin{array}{@{}rcl@{}} w(i) =\left\{ \begin{array}{c l} -\gamma/degree(i), & i \notin T\\ +\infty, & i \in T\\ \end{array}\right. \end{array} $$

where *w*(*i*) is the node weight of vertex *i*, *γ* is a positive constant value, *d* *e* *g* *r* *e* *e*(*i*) is the degree of vertex *i* in the protein-protein interaction network, and *T* is the compulsory terminal set. Note that, the degree centrality has been widely used to quantify the importance of vertices in networks [24], and proteins with low degrees are less likely to be important to cancer signal transduction. Furthermore, +*∞* ensures all the important proteins represented by compulsory terminals are contained in the identified subnetwork.

Node-weighted protein-protein interaction networks with these five types of elements can be generated using existing information on protein-protein interactions and cancer-related signaling pathways. An example is our generated node-weighted protein-protein interaction network for Homo sapiens (see Fig. 1). After the generation, we can use node-weighted Steiner tree techniques to identify subnetworks containing important elements of cancer-related signaling pathways.

### The modified node-weighted Steiner tree techniques

The node-weighted Steiner tree problem was separately proposed by Segev [25] and Duin [26] in 1987. It is a more general version of the classical Steiner tree problem in graphs. Since the classical Steiner tree problem in graphs is NP-hard, the node-weighted Steiner tree problem is also NP-hard, which means that there may not be an algorithm to solve large instances to optimality in polynomial time. Two types of Steiner tree techniques can deal with large networks efficiently. One is preprocessing technique, which makes large networks smaller and then easier to solve; the other one is heuristic algorithm, which finds suboptimal solutions in large networks in a short time. In this section, we first modify two preprocessing techniques to reduce sizes of node-weighted Steiner tree instances, then we modify a state-of-the-art heuristic algorithm for the prize-collecting Steiner tree problem to solve the node-weighted Steiner tree problem.

#### The modified preprocessing techniques

Many preprocessing techniques have been proposed for various Steiner tree problems [27, 28]. However, most of them cannot be used in networks with negative node weights, and thus cannot reduce sizes of node-weighted Steiner tree instances. In this subsection, we modify two preprocessing techniques to node-weighted Steiner tree instances.

**∙ Terminal degree 1 test:** *if |T|≥2, the edge adjacent to a compulsory terminal with degree 1 is in the optimal solution.*

The initial version of this test was proposed by Koch et al. in 1998 [29] to reduce sizes of classical Steiner tree instances. In the initial version, the condition |*T*|≥2 does not exist since it is implicitly met in all classical Steiner tree instances. However, in node-weighted Steiner tree instances, |*T*| may be 0 or 1. When |*T*|=1, the edge adjacent to a compulsory terminal with degree 1 may not be in the optimal solution. An example is a node-weighted Steiner tree instance where the optimal solution is the only compulsory terminal with degree 1. Therefore, by adding this condition, we modify this test to node-weighted Steiner tree instances.

**∙ Non-terminal degree 1 test:** *for any vertex i∉T with degree 1, if |T|≥1 and w(i)≤c(i,j) (vertex j is its adjacent vertex), then vertex i and edge (i,j) can be eliminated.*

The initial version of this test was proposed by Beasley in 1984 [30] to reduce sizes of classical Steiner tree instances. Nevertheless, this test cannot be applied to node-weighted Steiner tree instances without two conditions |*T*|≥1 and *w*(*i*)≤*c*(*i*,*j*). We modify this test to node-weighted Steiner tree instances by adding these two conditions.

The time complexity of these two modified techniques is *O*(|*V*|). Therefore, they can be conducted in large protein-protein interaction networks in a short time. Note that, more sophisticated preprocessing techniques can also be modified to node-weighted Steiner tree instances, and they may reduce instance sizes more significantly than these two techniques. However, sophisticated preprocessing techniques may be too slow in large protein-protein interaction networks. Hence, in this paper, we only modify these two simple techniques for our application. We leave the modification of more sophisticated preprocessing techniques to the future work.

#### The modified node-weighted Steiner tree algorithm

Many Steiner tree algorithms have been proposed in the last decades. However, most of them cannot be applied to networks with negative node weights, while the ones that can may be too slow to process large protein-protein interaction networks. In this subsection, we modify a fast implementation of the unrooted Goemans-Williamson algorithm proposed by Hegde et al. [20] in the 2014 DIMACS Implementation Challenge on Steiner tree problems (the initial version of this algorithm cannot be applied to networks with negative node weights). Our modified algorithm can be applied to networks with both positive and negative node weights, and it is fast to process large protein-protein interaction networks.

There are two phases in our modified algorithm: the growing phase and the pruning phase. In the growing phase, we use the “dynamic edge splitting” idea proposed by Cole et al. in 2001 [31] to find a raw solution tree in a short time. In the dynamic edge splitting process, we split each edge (*i*,*j*) into two edge parts *e* *p*(*i*,*j*) and *e* *p*(*j*,*i*). Let us define the edge splitting ratio *s* (*s*≥1) as follows.

3 $$\begin{array}{@{}rcl@{}} slack\{ep(i,j)\} =\left\{ \begin{array}{c l} c(i,j)/s, & i<j\\ (s-1)c(i,j)/s, & i>j \end{array}\right. \end{array} $$

where *s* *l* *a* *c* *k*{*e* *p*(*i*,*j*)} is the slack of edge part *e* *p*(*i*,*j*), *s* is a constant value and *s*≥1.

The two edge parts *e* *p*(*i*,*j*) and *e* *p*(*j*,*i*) share the slack (or cost) of edge (*i*,*j*) at the ratio of 1:(*s*−1), and they associate respectively with vertex *i* and *j*. The total number of edge parts is 2|*E*|, and the number of edge parts associated with each vertex equals to the degree of this vertex. An edge part is active when the vertex it associates with is in an active cluster, otherwise the edge part is inactive. Initially, we set each vertex as a cluster, and the slack of each cluster equals to its node weight. All the clusters with positive slacks are active. Note that, the slack of an inactive cluster may be negative.

All the active clusters and edge parts have their event time, which initially equals to their slacks. We maintain a global time value *t*_*g*_. As *t*_*g*_ increases, the slacks of active edge parts and clusters decrease. At any time, the remaining slack of an active cluster is the gap between its event time and *t*_*g*_; the remaining slack of an inactive cluster is the gap between its event time and its deactivation time; the remaining slack of an active edge part is the gap between its event time and *t*_*g*_; the remaining slack of an inactive edge part is the gap between its event time and the deactivation time of its cluster.

There are two types of events in the growing phase, which are the edge event and the cluster event, and they are triggered in the order of their event time. In the cluster event, we simply deactivate the corresponding cluster. In the edge event, the slack of the corresponding edge part becomes 0. Assume edge part *e* *p*(*i*,*j*) is the corresponding edge part for an edge event, and let *r* be the slack of edge part *e* *p*(*j*,*i*).

If *r*=0, then we merge the two clusters connected by edge (*i*,*j*) and their edge parts. The slack of new cluster equals to the sum of slacks of the two merged clusters. Suppose the slack of new cluster is *sl*, we set the event time of new cluster to be *t*_*g*_+*s* *l*. Note that, an inactive cluster may be merged into an active cluster in an edge event. In that case, we need to increase the event time of edge parts in the inactive cluster by the gap between *t*_*g*_ and its deactivation time. Furthermore, the most significant difference between our modified algorithm and its initial version is that the newly merged cluster may be inactive in our modified algorithm since the slack of the inactive cluster being merged may be negative.

If *r*>0, then we distinguish two cases to update the event time of these two edge parts:

Case 1: the cluster containing edge part *e* *p*(*j*,*i*) is active. Since we expect the slacks of these two edge parts to become 0 at the same time to trigger a merge event, we split the slack *r* evenly, and update the event time of both of these two edge parts to be *t*_*g*_+*r*/2.

Case 2: the cluster containing edge part *e* *p*(*j*,*i*) is inactive. We assume the cluster containing edge part *e* *p*(*j*,*i*) stays inactive until a merge event is triggered by edge (*i*,*j*). Then, we update the event time of *e* *p*(*i*,*j*) to be *t*_*g*_+*r*, and the event time of *e* *p*(*j*,*i*) to be the deactivation time of its cluster.

Note that, we update the event time of these two edge parts in the above way so that the two corresponding clusters would be merged in the next event on edge (*i*,*j*), assuming both clusters maintain their current activity status. If one of the two clusters changes its activity status, this will not hold. An extreme situation is that both clusters were active and the cluster containing edge part *e* *p*(*j*,*i*) becomes inactive since then. As a result, the next event on edge (*i*,*j*) will still have *r*>0, and we need to split the slack *r* again. In the worst case, the slack splitting case may keep happening endlessly. In this paper, we use a small value *μ* to deal with this case. If *r*<*μ*, we trigger the merge event. The optimization process of the growing phase terminates until there is no more than one active cluster left, and the subtree in the last active cluster is the raw solution tree we obtained in the growing phase. Note that, there may be no active cluster in the end of our growing phase, while there is always one active cluster left in the initial version of this algorithm.

In the pruning phase, we prune the raw solution tree above using the strong pruning algorithm proposed by Johnson et al. in 2000 [32]. In this pruning algorithm, we first attach each vertex with an *nw* value, which initially equals to its node weight. We define the processing degree of a vertex as the number of adjacent vertices that have not been processed. Initially, only leaves of the raw solution tree have a processing degree of 1. We randomly select a compulsory terminal to be the root. For non-root vertex *i* which has not been processed and whose processing degree is 1, assume vertex *j* is its adjacent vertex which has not been processed. If *c*(*i*,*j*)>*n* *w*(*i*), then we remove edge (*i*,*j*) and the subtree rooted at vertex *i*, or we update the *nw* value of vertex *j* using the following equation,

4 $$\begin{array}{@{}rcl@{}} {}nw(j)=nw(j)+nw(i)-c(i,j) \end{array} $$

We keep processing all the non-root vertices until all of them have been processed. The remaining subtree is the identified protein-protein interaction subnetwork. The steps of our modified algorithm are shown in Table 1. The time complexity of this algorithm is *O*(|*E*|*l* *o* *g*|*V*|). Thus, it is fast in large networks.

**Table 1:** The modified node-weighted Steiner tree algorithm

Input:	Protein-protein interaction network *G*, parameter *s*, *μ*
Output:	Subnetwork *T*_*r*_⊆*G*
1	Initialize *T*_*r*_=*∅*, global time *t*_*g*_, clusters, edge parts
2	**While** there are more than one active cluster **do**
3	Find the closest edge event time *t*_*e*_ and the responsible edge part *e* *p*_1_
4	Find the closest cluster event time *t*_*c*_ and the responsible cluster *C*
5	**If** *t*_*e*_≤*t*_*c*_ **then**
6	Update *t*_*g*_ to *t*_*e*_
7	Identify the corresponding edge part *e* *p*_2_ to *e* *p*_1_
8	**If** *e* *p*_1_ and *e* *p*_2_ are in the same cluster **then**
9	**continue**
10	**else**
11	Calculate *r*
12	**If** *r*>*μ* **then**
13	update the event time of *e* *p*_1_ and *e* *p*_2_
14	**else**
15	Add the corresponding edge to *T*_*r*_
16	Merge the two corresponding clusters and their edge parts
17	**else**
18	Update *t*_*g*_ to *t*_*c*_
19	Deactivate *C*
20	Remove the edges disconnected with the last active cluster from *T*_*r*_
21	Associate each vertex in *T*_*r*_ with an *nw* value
22	Randomly select a compulsory terminal as the root of *T*_*r*_
23	**While** not all the non-root vertices in *T*_*r*_ have been processed**do**
24	**For** unprocessed non-root vertex *i* whose processing degree is 1 **do**
25	Find the unprocessed adjacent vertex *j*
26	**If** *c*(*i*,*j*)>*n* *w*(*i*)**then**
27	Remove edge (*i*,*j*) and the subtree rooted at vertex*i* from *T*_*r*_
28	**else**
29	Update *n* *w*(*j*) using Eq. (4)
30	Mark vertex *i* as processed

### New metrics for the selection of important elements

We use node-weighted Steiner tree techniques to identify a protein-protein interaction subnetwork. After the identification, we evaluate the importance of proteins and protein-protein interactions in it. The important ones are selected as the identified important elements of cancer-related signaling pathways.

There are many metrics to evaluate the importance of network elements, among which betweenness centrality is the most popular one [33]. The original betweenness centrality was proposed by Bavelas in 1948 [34]. He suggested that in a group of people, the person who is strategically located on the shortest communication path connecting pairs of others is considered important since he can influence the group by withholding, coloring or distorting information. Nevertheless, the original betweenness centrality assumes signals transduce evenly between each pair of vertices, while in cancer-related signaling pathways, signals mainly transduce from source to terminal proteins. Thus, the original betweenness centrality fails to consider different functions of proteins in cancer-related signaling pathways. In this section, we propose two new metrics to evaluate the importance of proteins and protein-protein interactions in the identified subnetwork. These new metrics overcome the weakness of the original betweenness centrality by only considering signals transducing between source and terminal proteins.

Let *S* and *T*′ be respectively the sets of source and terminal proteins of cancer-related signaling pathways, then we define the betweenness degree of protein *m* as

5 $$ B(m) = \sum_{i \in S, j \in T\prime} SP_{ij}(m)  $$

where *S* *P*_*ij*_ is the shortest path between source protein *i* and terminal protein *j* in the identified subnetwork (since the identified subnetwork is always a tree, there is only one shortest path between *i* and *j*), and *S* *P*_*ij*_(*m*)=1 if protein *m* is in this path, or *S* *P*_*ij*_(*m*)=0. A protein with a high betweenness degree is considered important.

Similar to betweenness degree of proteins, we define the betweenness degree of protein-protein interaction *e*_*mn*_ as

6 $$ B(e_{mn}) = \sum_{i \in S, j \in T\prime} SP_{ij}(e_{mn})  $$

where *e*_*mn*_ is the interaction between protein *m* and protein *n*, *S* *P*_*ij*_(*e*_*mn*_)=1 if *e*_*mn*_ is in *S* *P*_*ij*_, or *S* *P*_*ij*_(*e*_*mn*_)=0. A protein-protein interaction with a high betweenness degree is considered important. The following inequality is always met.

7 $$ B(m) \geq B(e_{mn}) |\, \forall m,n \in V  $$

Therefore, proteins connected by interactions with high betweenness degrees will also have high betweenness degrees, which is reasonable since proteins connected by important interactions are important too.

Calculating betweenness degrees needs to find the shortest path multiple times. Since the time complexity of finding the shortest path is *O*(|*V*|^2^) [35], it is tremendously slow to apply these new metrics directly to large node-weighted protein-protein interaction networks (even though they are much faster than the original betweenness centrality). On the contrary, since the identified subnetwork is often small (for example, there are only 29 proteins and 28 protein-protein interactions in the identified subnetwork in our generated node-weighted protein-protein interaction network for Homo sapiens), it is fast to calculate betweenness degrees of all the proteins and protein-protein interactions in the identified subnetwork. After the calculation, we select the ones with high betweenness degrees as the identified important elements of cancer-related signaling pathway. A further experimental-evidence-based analysis can be conducted on them.

## Results

The PI3K/Akt and MAPK signaling pathways are widely known to account for the causes of various cancers [36–38]. Nevertheless, the existing information on them may not be complete. Therefore, in this section, we apply our node-weighted Steiner tree approach to identify their important elements. After the identification, we analyze the roles of the identified elements in cancer signal transduction by exploring previously reported experimental evidences.

### Application to identify important elements of PI3K/Akt and MAPK signaling pathways

First, we generate a node-weighted protein-protein interaction network using existing information on protein-protein interactions and PI3K/Akt and MAPK signaling pathways. There are many databases on protein-protein interactions, such as BIND [39], BioGRID [40], DIP [41], OPHID [42] and String [43]. Similarly, there are many databases on signaling pathways, such as KEGG [4], Reactome [44], PANTHER [45], and Pathway Commons [46]. Since String is one of the most comprehensible databases of protein-protein interactions (there are 2031 organisms, 9.6 million proteins, and 184 million protein-protein interactions in String to date) and KEGG is one of the most comprehensible databases of signaling pathways [47], we use String and KEGG data to generate the node-weighted protein-protein interaction network.

**Fig. 2:**
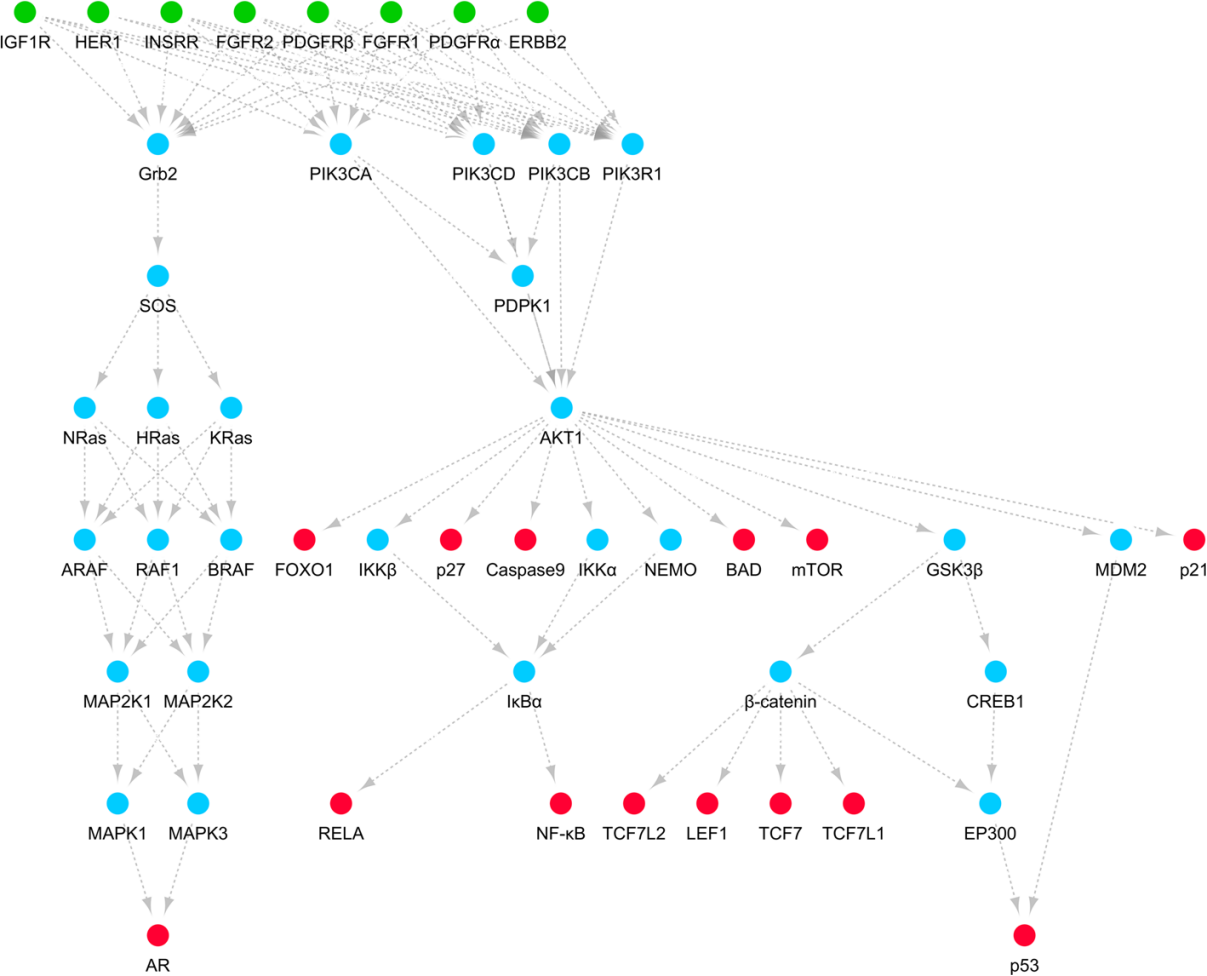
The protein-based PI3K/Akt and MAPK signaling pathways in KEGG. The green and red nodes respectively represent source and terminal proteins for cancer signal transduction, while the blue nodes represent junction proteins. These signaling pathways are generated by transforming genes and genomes in the signaling pathways in KEGG to the corresponding proteins. They are used to further generate our node-weighted protein-protein interaction network

String data can be directly used in the generation process. On the contrary, KEGG data cannot be directly used since it is recorded at the level of genes and genomes, not at the level of proteins. We need to transform the genes and genomes in the PI3K/Akt and MAPK signaling pathways in KEGG to the corresponding proteins. After the transformation, we obtain the PI3K/Akt and MAPK signaling pathways at the level of proteins, which are shown in Fig. 2. Note that, only protein-protein interactions that are justified by the experimental evidences in String are recorded in them. Moreover, these KEGG pathways may not be complete. Evidences of their unknown elements may exist in String, but not in KEGG. Thus, the identification of their important elements still needs to be conducted in our node-weighted protein-protein interaction network, which is generated using both String and KEGG data.

Our node-weighted protein-protein interaction network contains proteins in the full collection of Homo sapiens data in String, where protein-protein interactions are recorded based on multiple types of evidences. We select the protein-protein interactions based on experimental evidences to generate edges in this network. Note that, these experimental evidences record multiple types of protein-protein interactions, such as protein binding and transcription regulation. The parameters to generate edge costs and node weights are *α*=2×10^6^, *β*=2, *γ*=5. Note that, *con* is the experimental score in String that reflects the confidence of the existence of protein-protein interactions. Since protein-protein interactions in the PI3K/Akt and MAPK signaling pathways in KEGG are more likely to exist and be important, we increase their confidence scores by 50% while calculating edge costs. Moreover, in the PI3K/Akt and MAPK signaling pathways, signals transduce from source proteins to terminal proteins. Since these source and terminal proteins (see Fig. 2) are well known to be important to cancer signal transduction, we mark them as compulsory terminals. There are 22 compulsory terminals in total. The topology of our generated node-weighted protein-protein interaction network is illustrated in Fig. 1. There are 16,843 vertices and 1,736,922 edges in total. On a commonly used personal computer with a 4.2 GHz i7-7700K CPU, the running time of its generation is around 1.5 s (excluding the running time to input String and KEGG data).

After the generation, we apply our modified node-weighted Steiner tree techniques to identify a subnetwork. On the same computer, the running time of our modified preprocessing techniques and node-weighted Steiner tree algorithm are respectively 0.003 and 0.05 second. Our modified preprocessing techniques reduce the size of our node-weighted protein-protein interaction network to 15,715 vertices and 1,735,794 edges, which is significant when considering their short running time.

**Fig. 3:**
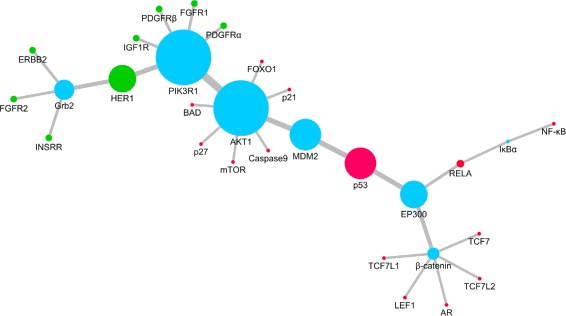
The identified protein-protein interaction subnetwork. The diameters of nodes and widths of edges are in scale with the betweenness degrees of the corresponding proteins and protein-protein interactions

The identified subnetwork, which is shown in Fig. 3, contains important elements of PI3K/Akt and MAPK signaling pathways. All the proteins and most of the protein-protein interactions in the identified subnetwork are already in the PI3K/Akt and MAPK signaling pathways in KEGG (see Fig. 2). However, two protein-protein interactions ((EP300, RELA) and (*β*-catenin, AR)) in the identified subnetwork are not in these KEGG pathways. These newly identified protein-protein interactions may also be important to cancer signal transduction (an experimental-evidence-based analysis is later conducted on them).

To select important elements of PI3K/Akt and MAPK signaling pathways from the identified subnetwork, we calculate betweenness degrees of all the proteins and protein-protein interactions in it using Eqs. (5) and (6). The results are shown in Tables 2 and 3. On the same computer, the running time of the calculation process is around 0.3 s. Since 8 source proteins and 14 terminal proteins are distinguished in the calculation process, we set 14 as the threshold value, and select proteins and protein-protein interactions with a betweenness degree larger than 14 as the identified important elements of PI3K/Akt and MAPK signaling pathways.

**Table 2:** The betweenness degrees of proteins in the identified subnetwork

Protein	Betweenness	Protein	Betweenness	Protein	Betweenness
**AKT1**	**112**	PDGFR *β*	14	LEF1	8
**PIK3R1**	**112**	IGF1R	14	TCF7L1	8
**p53**	**64**	ERBB2	14	BAD	8
**MDM2**	**64**	INSRR	14	Caspase9	8
**EP300**	**56**	FGFR1	14	TCF7	8
**HER1**	**56**	FGFR2	14	mTOR	8
**Grb2**	**42**	I *κ* *B* *α*	8	AR	8
***β*** **-catenin**	**40**	NF- *κ*B	8	FOXO1	8
**RELA**	**16**	p27	8	TCF7L2	8
PDGFR *α*	14	p21	8		

The bold font is used to highlight the identified important proteins of PI3K/Akt and MAPK signaling pathways

**Table 3:** The betweenness degrees of protein-protein interactions in the identified subnetwork

Protein 1	Protein 2	Betweenness	Protein 1	Protein 2	Betweenness
**AKT1**	**PIK3R1**	**112**	Grb2	FGFR2	14
**AKT1**	**MDM2**	**64**	I *κ* *B* *α*	NF- *κ*B	8
**p53**	**MDM2**	**64**	I *κ* *B* *α*	RELA	8
**EP300**	**p53**	**56**	p27	AKT1	8
**PIK3R1**	**HER1**	**56**	p21	AKT1	8
**HER1**	**Grb2**	**42**	LEF1	*β*-catenin	8
**EP300**	***β*** **-catenin**	**40**	AKT1	BAD	8
**EP300**	**RELA**	**16**	AKT1	Caspase9	8
PDGFR *α*	PIK3R1	14	AKT1	mTOR	8
PDGFR *β*	PIK3R1	14	AKT1	FOXO1	8
IGF1R	PIK3R1	14	TCF7L1	*β*-catenin	8
ERBB2	Grb2	14	TCF7	*β*-catenin	8
PIK3R1	FGFR1	14	*β*-catenin	AR	8
Grb2	INSRR	14	*β*-catenin	TCF7L2	8

The bold font is used to highlight the identified important protein-protein interactions of PI3K/Akt and MAPK signaling pathways

### Analysis of the identified important elements of PI3K/Akt and MAPK signaling pathways

There are 9 proteins and 8 protein-protein interactions (the ones that are marked in bold in Tables 2 and 3) that have been identified as important elements of PI3K/Akt and MAPK signaling pathways. We analyze their roles in cancer signal transduction by exploring previously reported experimental evidences.

The PI3K/Akt pathway contributes to tumorigenesis of various cancers by regulating cell cycles, survival, growth and proliferation [48]. In brief, PI3K, as the downstream of growth factor receptor tyrosine kinases (RTKs), catalyzes Phosphatidylinositol(3,4,5)-trisphosphate (PIP3) to activate the downstream molecular Akt. Previous experiments have shown that all RTKs have the ability to activate the PI3K/Akt pathway [49]. Nevertheless, our identification indicates HER1 plays a major role in them. As a matter of fact, Akt isoforms also play important roles in the activation of PI3K/Akt pathway [50]. Our identification confirms that Akt1 is a key factor in Akt family as well as the whole PI3K/Akt pathway. Interestingly, PI3KR1 has been identified as important as Akt1, which suggests it may be responsible for most protein-protein interactions of PI3K [51]. On the other hand, TP53, as a common tumor suppressor gene, was widely found to be mutant in many cancers [52]. Thus, the identification of p53 indicates the PI3K/Akt pathway affects cells mainly by inhibiting p53 and then inducing the loss of cell cycles control. As an inhibitor of p53 [53], it is unsurprising that MDM2 has also been identified as important. Similarly, the identification of EP300, a negative regulator of p53, confirms the significance of p53 to the PI3K/Akt pathway. Furthermore, since *β*-catenin affects p53 by inactivating EP300 [54], it is understandable that it has also been identified as important. Remarkably, we have identified the interaction between EP300 and RELA as important, even though it is not in the PI3K/Akt pathway in KEGG. Recent experiments have shown the existence of this interaction in cancer signal transduction [55, 56], while our identification indicates that this interaction may induce a even stronger crosstalk between p53 and NF- *κ*B pathway than we had expected. Moreover, its identification provides a theoretical support for previous discovery that p53 has an effect on the activation of NF- *κ*B pathway after irradiation [57]. Ultimately, Grb2, which mediates RTKs and SOS [58], is the only protein that has been identified in the MAPK signaling pathway, which indicates the MAPK signaling pathway may play a less significant role in cancer signal transduction than the PI3K/Akt pathway.

In summary, the significance of most of these identified elements to the PI3K/Akt and MAPK signaling pathways have already been indicated by previous experimental evidences. Nevertheless, our identification provides a deeper understanding towards them. Moreover, new findings are indicated in this process, such as the strong crosstalk between p53 and NF- *κ*B pathway that may be underestimated before. To ensure our predications are real, new experiments are suggested to conduct in the future, such as the ones using the Co-immunoprecipitation technique [59] to identify physiologically relevant protein-protein interactions.

## Discussion

In this paper, we propose the node-weighted Steiner tree approach to identify important elements of cancer-related signaling pathways at the level of proteins. This new approach is fast in processing large protein-protein interaction networks. Moreover, it overcomes the weaknesses of previous Steiner tree approaches by attaching vertices with both positive and negative node weights.

Since the PI3K/Akt and MAPK signaling pathways are well known to account for the causes of various cancers, we take them as an example, and apply our approach to identify their important elements. We first generate a node-weighted protein-protein interaction network. There are five types of elements in this network, which are vertex, edge, compulsory terminal, edge cost, and node weight. Each vertex represents a protein; each edge represents a protein-protein interaction; each compulsory terminal represents an important protein to cancer signal transduction; each edge cost represents an in-confidence score of the existence of protein-protein interaction; each node weight represents a confidence score of the existence of protein in cancer-related signaling pathways. Under these representations, we can identify a subnetwork containing important elements of PI3K/Akt and MAPK signaling pathways by solving the node-weighted Steiner tree problem.

Since String and KEGG are the most comprehensible databases of protein-protein interactions and signaling pathways, we use String and KEGG data to generate this network. After the generation, we use Steiner tree techniques to identify a subnetwork in it. Most existing Steiner tree techniques cannot be applied to networks with negative node weights, while the ones that can may be too slow in large protein-protein interaction networks. Two types of Steiner tree techniques can deal with large networks efficiently, which are preprocessing technique and heuristic algorithm. Therefore, we first modify two preprocessing techniques to reduce sizes of node-weighted Steiner tree instances. Then, we modify a state-of-the-art heuristic algorithm for the prize-collecting Steiner tree problem to solve the node-weighted Steiner tree problem. Our modified algorithm can be applied to networks with both positive and negative node weights, and it is fast in large protein-protein interaction networks. We apply our modified Steiner tree techniques to identify a subnetwork in our generated node-weighted protein-protein interaction network.

Subsequently, we use network evaluation metrics to evaluate the importance of proteins and protein-protein interactions in the identified subnetwork. Betweenness centrality is widely used to evaluate the importance of vertices and edges in networks. However, the original betweenness centrality assumes signals transduce evenly between each pair of vertices, while in cancer-related signaling pathways, signals mainly transduce from source to terminal proteins. Hence, the original betweenness centrality fails to consider different functions of proteins in cancer-related signaling pathways. In this paper, we propose two new metrics to evaluate the importance of proteins and protein-protein interactions. These new metrics overcome the weakness of the original betweenness centrality by only considering signals transducing between source and terminal proteins. We use them to calculate betweenness degrees of all the proteins and protein-protein interactions in the identified subnetwork. Then, we select the ones with high betweenness degrees as the identified important elements of PI3K/Akt and MAPK signaling pathways. A further experimental-evidence-based analysis is conducted to demonstrate their significance to cancer signal transduction.

### Parameter settings in the generation of node-weighted protein-protein interaction network

There are three parameters in the generation of node-weighted protein-protein interaction network, which are *α*,*β* in Eq. (1) and *γ* in Eq. (2). *α*,*β* determine the values of edge costs, while *γ* determines the values of node weights of non-compulsory proteins. We first set the value of *β*. For two edges *e*_1_,*e*_2_, if their confidence scores (refer to Eq. (1)) are respectively $con_{e_{1}},con_{e_{2}}$, then the ratio of their edge costs (*e*_1_ to *e*_2_) is $(con_{e_{2}}/con_{e_{1}})^{\beta }$. Thus, a small *β* induces a small variance between edge costs, while a large *β* induces a large variance between edge costs. Since the experimental scores in String reflect, but not accurately reflect the confidence of the existence of protein-protein interactions, it is not recommended to set *β* too small or too big. In this paper, we set *β*=2 to show our “moderate” confidence in these scores. After *β*, we set the value of *α*, which does not affect the ratios of costs of different edges. From the aesthetic perspective, we set *α*=2×10^6^ to make the edge costs distributed around 100 to 300. After *α*,*β*, we set the value of *γ*. A small *γ* gives small negative node weights to non-compulsory proteins, while a large *γ* gives large negative node weights to them. Hence, a small *γ* may result in the identification of unrelated proteins, while a large *γ* may result in the missed identification of proteins in possible new cancer-related signaling pathways. In this paper, we set *γ*=5 to make a balance between removing unrelated proteins and keeping interested ones. Furthermore, we apply our node-weighted Steiner tree approach multiple times with different values of *β*,*γ* (*α* is fixed at 2×10^6^), and the resulting percentages of identified proteins that are in the PI3K/Akt and MAPK signaling pathways in KEGG are shown in Table 4. It can be seen that *β* has a bigger impact on the identification result than *γ*, and with the parameter settings above, the identification result overlaps the most with KEGG data.

**Table 4:** The percentages of identified proteins that are in the PI3K/Akt and MAPK signaling pathways in KEGG

	*γ*=1	*γ*=5	*γ*=10	*γ*=100	*γ*=1000	*γ*=10000
*β*=1	74.07%	74.07%	74.07%	74.07%	74.07%	74.07%
*β*=2	92.86%	92.86%	92.86%	92.86%	82.14%	82.14%
*β*=3	89.29%	82.14%	82.14%	82.14%	82.14%	82.14%

### Advantages of our node-weighted Steiner tree approach

We aim to identify important elements of cancer-related signaling pathways at the level of proteins. There are mainly three types of approaches to identify signaling pathways for human diseases, which are the experimental approach, the systematic approach, and the data-driven approach. Protein-protein interaction networks are often very large, and only the data-driven approach is fast enough to process them. The Steiner tree approach is an efficient data-driven approach. Two types of Steiner tree approaches have already been applied to biomedical networks, which are the classical Steiner tree approach and the prize-collecting Steiner tree approach. However, as to protein-protein interaction networks, the classical Steiner tree approach fails to consider the properties of different proteins, while the prize-collecting Steiner tree approach may identify irrelevant proteins. Therefore, neither of them is suitable for processing protein-protein interaction networks. On the contrary, our node-weighted Steiner tree approach advantages these two approaches since it considers the properties of different proteins by attaching them with node weights and it can avoid irrelevant proteins by attaching them with negative node weights.

Furthermore, our node-weighted Steiner tree approach is fast in processing protein-protein interaction networks. To our knowledge, our generated node-weighted protein-protein interaction network is the largest single protein-protein interaction network that has ever been generated and analyzed as a whole. Even so, on a commonly used personal computer, our approach only takes less than 2 seconds to identify important elements of PI3K/Akt and MAPK signaling pathways (the running time to generate this large node-weighted protein-protein interaction network is also included). Especially, our modified node-weighted Steiner tree algorithm only takes around 0.05 s to identify a subnetwork. As a matter of fact, this algorithm is still fast in much larger networks. The running time of this algorithm in three networks of different sizes is shown in Table 5, in which the PPI network is our generated node-weighted protein-protein interaction network; the Hand network is a network generated by others for image processing [20]; the M network is a network randomly generated by ourselves. All the experiments are conducted on a commonly used personal computer with a 4.2 GHz i7-7700K CPU. It can be seen that our modified node-weighted Steiner tree algorithm is still reasonably fast in the largest network with 1 million vertices and 10 millions edges. Therefore, our node-weighted Steiner tree approach can be used to process large biomedical networks in scenarios where fast computation is required. Moreover, the speed of our algorithm, which is fast enough to process thousands of networks in a reasonable amount of time, opens up the possibility of an exploratory approach in which putative new source nodes (e.g. proteins of genes with recurrent mutations in cancer) are explored in turn to search for possible novel cancer drivers.

**Table 5:** The running time of our modified node-weighted Steiner tree algorithm in networks of different sizes

Network	PPI	Hand	M
|*V*|	16,843	158,400	1,000,000
|*E*|	1,736,922	315,808	10,000,000
Running time	0.05s	0.3s	30s

## Conclusion

Cancer is a major health problem in our society. A complete understanding of cancer-related signaling pathways will greatly benefit its prevention, diagnosis, and treatment. In this work, we propose the node-weighted Steiner tree approach to identify important elements of cancer-related signaling pathways at the level of proteins. In this new approach, we first generate a node-weighted protein-protein interaction network using existing information on protein-protein interactions and cancer-related signaling pathways. Then, we modify two preprocessing techniques and a state-of-the-art Steiner tree algorithm to identify a subnetwork in it. After that, we propose two new metrics to select important elements of cancer-related signaling pathways from this subnetwork. We apply this new approach to identify important elements of two well-known cancer-related signaling pathways: the PI3K/Akt and MAPK signaling pathways. On a commonly used personal computer, this new approach takes less than 2 seconds to identify their important elements in the full-scale node-weighted protein-protein interaction network for Homo sapiens. We analyze and demonstrate the significance of these identified elements to cancer signal transduction by exploring previously reported experimental evidences. A deeper understanding towards the PI3K/Akt and MAPK signaling pathways is gained in this process. In conclusion, our node-weighted Steiner tree approach is shown to be both fast and effective to identify important elements of cancer-related signaling pathways. Hence, it can be applied to areas where fast processing of large protein-protein interaction data is required.
